# Buffalo Academy of Medicine

**Published:** 1902-10

**Authors:** F. W. McGuire


### SOCIETY PROCEEDINGS.

### Buffalo Academy of Medicine.

REPORTED BY F. W. McGUIRE, M. D., Secretary.

*Stated Meeting, September 9, 1902.*

The meeting was called to order by the President, Dr. HERMAN E. HAYD at 9 p.m.

The minutes of the last stated meeting, held May 20, 1902, were read and approved.

The council recommended the election of Dr. Edgar R. McGuire for resident membership. He was duly balloted for and elected a member of the Academy. The council also recommended the election of Dr. Eugene A. Smith as trustee to fill out the unexpired term of Dr. Howell.

It was moved and seconded that Dr. Smith be nominated for the office of trustee to fill the vacancy caused by Dr. Howell's resignation, and on motion the secretary was directed to cast a ballot for Dr. Smith, who was then declared elected.

The program of the evening was provided by the Section of Medicine. It consisted of a paper read by Dr. FLOYD S. CREGO entitled,

SYPHILIS OF THE NERVOUS SYSTEM.

A paper was also read by Dr. SYDNEY A. DUNHAM, entitled,

CEREBRAL HEMORRHAGE FOLLOWING INJURY TO THE SPINE.

The papers were freely discussed by Drs. Blaauw, Frost, Dunham and Crego.

Adjourned at 10.20 p.m.

